# Mitophagy in neurodegeneration and aging

**DOI:** 10.3389/fgene.2012.00297

**Published:** 2012-12-19

**Authors:** Konstantinos Palikaras, Nektarios Tavernarakis

**Affiliations:** Institute of Molecular Biology and Biotechnology, Foundation for Research and Technology - Hellas, HeraklionCrete, Greece

**Keywords:** aging, autophagy, neuron, mitochondria, mitophagy, neurodegeneration, parkin, PINK1

## Abstract

Macroautophagy is a cellular catabolic process that involves the sequestration of cytoplasmic constituents into double-membrane vesicles known as autophagosomes, which subsequently fuse with lysosomes, where they deliver their cargo for degradation. The main physiological role of autophagy is to recycle intracellular components, under conditions of nutrient deprivation, so as to supply cells with vital materials and energy. Selective autophagy also takes place in nutrient-rich conditions to rid the cell of damaged organelles or protein aggregates that would otherwise compromise cell viability. Mitophagy is a selective type of autophagy, whereby damaged or superfluous mitochondria are eliminated to maintain proper mitochondrial numbers and quality control. While mitophagy shares key regulatory factors with the general macroautophagy pathway, it also involves distinct steps, specific for mitochondrial elimination. Recent findings indicate that parkin and the phosphatase and tensin homolog-induced putative kinase protein 1 (PINK1), which have been implicated in the pathogenesis of neurodegenerative diseases such as Parkinson’s disease, also regulate mitophagy and function to maintain mitochondrial homeostasis. Here, we survey the molecular mechanisms that govern the process of mitophagy and discuss its involvement in the onset and progression of neurodegenerative diseases during aging.

## INTRODUCTION

Macroautophagy (henceforth referred to as autophagy) is a high-regulated catabolic process responsible for the lysosomal degradation of cytoplasmic constituents. The main characteristic of the autophagic pathway is the formation of a double-membrane structure known as autophagosome, which engulfs cytoplasmic cargo and delivers it to lysosomes for degradation ( Klionsky, 2007). In direct correlation with the large variety of autophagy substrates, including cytoplasmic proteins, ribosomes, organelles, bacteria and viruses, autophagy defects have been associated with a wide range of human disorders, such as cancer, autoimmune and neurodegenerative diseases ( Mizushima et al., 2008). The main physiological role of autophagy is to supply the cell with essential materials and energy by recycling intracellular components, under conditions of nutrient deprivation when nutrients cannot be obtained from the extracellular environment. Selective types of autophagy, including pexophagy ( Sakai et al., 2006), ribophagy ( Kraft et al., 2008), ER-phagy ( Bernales et al., 2007), protein selective chaperone-mediated autophagy ( Cuervo et al., 2004), nucleophagy ( Mijaljica et al., 2010), mitochondrial autophagy (mitophagy; Lemasters, 2005) take place under nutrient-rich conditions to rid the cell of damaged organelles or protein aggregates that would otherwise compromise cell viability.

Mitochondria are double-membrane-bound organelles, essential for energy production and cellular homeostasis in eukaryotic cells. In addition, mitochondria have vital roles in calcium signaling and storage, metabolite synthesis, and apoptosis ( Kroemer et al., 2007). Thus, mitochondrial biogenesis, as well as, elimination of damaged and superfluous mitochondria are highly regulated processes. Mitophagy is a selective type of autophagy that mediates the removal of mitochondria. Through mitophagy cells regulate mitochondrial number in response to their metabolic state and also implement a quality control system for proper elimination of damaged mitochondria. The process of mitophagy is highly regulated and conserved from yeast to mammals (**Table 1**). While mitophagy shares key regulatory factors with the general autophagy pathway, it also involves distinct steps, specific for mitochondrial elimination. Studies in yeast identified specific genes that are required for mitophagy, but not for other types of autophagy ( Kanki et al., 2009a; Kanki and Klionsky, 2010), demonstrating the selective regulation of this process. Despite the fact that the actual selection of mitochondria for degradation is a still obscure part of the process, recent studies shed light on the mechanisms that govern mitophagy and regulate removal of mitochondria during developmental processes or upon mitochondrial damage. In this review, we survey the molecular mechanisms that mediate mitophagy and also highlight how defects in this process may contribute to the onset and progression of neurodegenerative diseases during aging.

**Table 1:** Mitophagy-specific factors are highly conserved form yeast to mammals.

Organism				Function	Role
*Saccharomyces cerevisiae*	*Caenorhabditis elegans*	*Drosophila melnogaster*	*Mus musculus*
Atg32	–	–	–	Mitophagy receptor	Interaction with Atg8 recruits the autophagic machinery
–	DCT-1	–	NIX/BNIP3	Mitophagy receptor	Interaction with LC3/GABARAP recruits the autophagic machinery
–	PINK-1	Pink1	PINK1	Ser/Thr protein kinase	Phosphorylates and recruits Parkin to mitochondria
–	PDR-1	Parkin	PARKIN	E3 ubiquitin ligase	Ubiquitinates outer membrane mitochondrial proteins such as Mfn1/2, VDAC, MIRO1/2
–	SQST-1 (T12G3.1)	Ref(2)P	SQST-1/p62	Adaptor protein	Interacts with ubiquitinated proteins to recruit the autophagic machinery
Fzo1	FZO-1	Fzo, Dmfn	MFN-1/2	Outer membrane fusion	Ubiquitinated by Parkin; their degradation precedes mitophagy induction
Vdac1	VDAC-1 (R05G6.7)	DmVDAC	VDAC1	Voltage-dependent anion channel; outer mitochondrial membrane	Upon ubiquitination by Parkin induces the recruitment of the autophagic machinery

## MOLECULAR MECHANISMS OF MITOPHAGY

The molecular mechanisms of mitophagy were studied in the yeast *Saccharomyces cerevisiae*. The yeast *uth1* gene encodes a Sad1p/UNC-84 (SUN)-domain protein that is located in the outer mitochondrial membrane and is essential for the specific autophagic elimination of mitochondria upon nitrogen starvation or rapamycin treatment, without influencing general autophagy ( Kissova et al., 2004). The protein Aup1, a member of protein phosphatase 2C (PP2C) superfamily that is located in the mitochondrial intermembrane space, is essential for efficient mitophagy at the stationary phase ( Tal et al., 2007). Aup1 may regulate mitophagy by also controlling the retrograde response pathway ( Journo et al., 2009).

**FIGURE 1:**
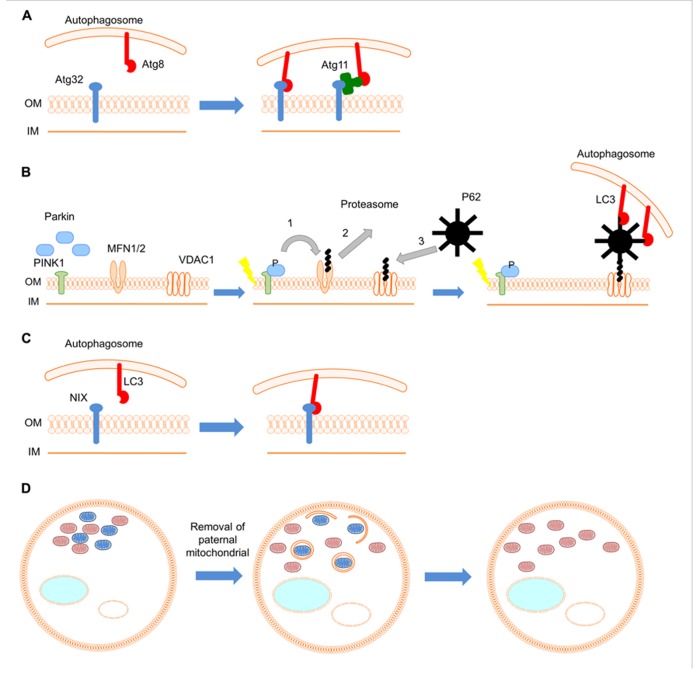
**Mechanisms and roles of mitophagy**. **(A)** In yeast Atg32 (blue), a mitochondrial outer membrane protein, interacts with Atg8 (red) directly or indirectly through the adaptor protein Atg11 (green), and links mitochondria to autophagic machinery. **(B)** During red blood cell development the mitochondrial population is eliminated by mitophagy. Nix (blue), an outer mitochondrial membrane protein, serves as a receptor for targeting mitochondria to autophagosomes through its interaction with the autophagosomal protein LC3 (red). **(C)** In the fertilized *C. elegans *embryo, the autophagic pathway selectively degrades sperm-derived mitochondria (blue; oocyte-derived mitochondria are shown in pink). **(D)** Upon mitochondrial depolarization, PINK1 (green) is stabilized on the outer mitochondrial membrane. Subsequently, Parkin (blue) is recruited and ubiquitylates outer mitochondrial membrane proteins such as MFN1/2 and VDAC1. (1) Ubiquitinated MFN1/2 is degraded by the proteasome system. Damaged mitochondria are isolated and cannot fuse with the healthy mitochondrial population. (2) Next, ubiquitin-binding adaptor molecules, such as p62 (black), are recruited to mitochondria to initiate mitophagy through their interaction with LC3 (red).

Another factor required for mitophagy is Atg32, a 59 kDa protein, located in the outer mitochondrial membrane ( Kanki et al., 2009b; Okamoto et al., 2009a). The amino- and carboxy-terminal domains of Atg32 are oriented toward the cytoplasm and intermembrane space, respectively. Atg32 is thought to act as a mitochondrial receptor that binds the adaptor protein Atg11, to sequester mitochondria to the phagophore assembly site (PAS), during mitophagy ( Okamoto et al., 2009b). The cytosolic domain of Atg32 contains an evolutionary conserved WXXL-like motif, which is critical for the interaction with Atg8 (the yeast homolog of the mammalian autophagosome protein LC3; Okamoto et al., 2009b). Thus, Atg32 can interact with Atg8 directly through the WXXL-like motif or indirectly through Atg11. This association is thought to recruit autophagosomes to mitochondria (**Figure 1A**). Atg32 is the first protein shown to interact with the core autophagic machinery, and be required specifically for mitophagy. Interestingly, loss of Atg32 does not alter cellular reactive oxygen species (ROS) levels or growth on non-fermentable carbon sources ( Kanki et al., 2009b). This suggests the existence of additional Atg32-independent mitophagy pathways. Recent studies identified two mitogen-activated protein kinases (MAPKs), Stl2 and Hog1, also required for the specific elimination of mitochondria via autophagy in *S. cerevisiae *( Mao et al., 2011). These two positive regulators establish an additional regulatory step in the process of mitophagy, underlining the complexity of this organelle quality control system.

### THE PINK1/PARKIN PATHWAY IN MITOPHAGY REGULATION

Mutations in the genes encoding the cytosolic E3 ubiquitin ligase Parkin and the mitochondrial phosphatase and tensin homolog (PTEN)-induced kinase 1 (PINK1) have been shown to cause a recessive form of parkinsonism ( Kitada et al., 1998; Valente et al., 2004). However, the involvement of these proteins in the pathogenesis of Parkinson’s disease remained obscure. Studies in *Drosophila melanogaster *indicate that PINK1 and Parkin act in the same genetic pathway to regulate mitochondrial network integrity ( Greene et al., 2003; Park et al., 2006). In healthy mitochondria, PINK1 is probably imported through the translocase complexes of the outer and inner mitochondrial membrane (TOM and TIM, respectively). PINK1 is subsequently cleaved by several proteases such as the mitochondrial-processing protease (MPP), the inner membrane presenilin-associated rhomboid-like protease (PARL; Meissner et al., 2011; Greene et al., 2012). Upon mitochondrial depolarization, import of PINK1 to the inner mitochondrial membrane is blocked and PINK1 is stabilized on outer mitochondrial membrane ( Lazarou et al., 2012). Accumulation of PINK1 on the mitochondrial surface induces mitophagy by recruiting Parkin to damaged mitochondria through a mechanism that is not well-understood. Thus, PINK1 likely functions as a sensor for damaged mitochondria. Recent studies have demonstrated that translocation of Parkin to impaired mitochondria requires PINK1 activity ( Narendra et al., 2008; Geisler et al., 2010; Matsuda et al., 2010; Narendra et al., 2010; Vives-Bauza et al., 2010). Following translocation, Parkin ubiquitylates outer mitochondrial membrane proteins. Subsequently other adaptor molecules, such as p62, are recruited to mitochondria to initiate mitophagy (**Figure 1D**). The mitochondrial fusion proteins mitofusin 1 and 2 have been identified as substrates of Parkin ( Gegg et al., 2010; Poole et al., 2010; Tanaka et al., 2010; Rakovic et al., 2011). Parkin prevents mitochondrial fusion through degradation of mitofusins, thereby isolating impaired mitochondria from the healthy mitochondrial population. Apart from mitofusins, overexpression of Parkin also mediates the ubiquitination of other outer mitochondrial membrane proteins, such as the voltage-dependent anion channel (VDAC), the mitochondrial Rho GTPases (MIRO) 1 and 2, as well as components of mitochondrial translocase complex (TOM70, TOM40, and TOM20; Chan et al., 2011; Yoshii et al., 2011). However, the relevance of these substrates to the induction of mitophagy *in vivo *remains to be investigated.

### THE ROLE OF MITOPHAGY IN DEVELOPMENT

Certain developmental processes entail removal of non-damaged mitochondria, a process that is essential for successful organ and tissue development. During erythrocyte differentiation, mitophagy eliminates healthy mitochondria in programmed fashion. Erythrocytes transfer oxygen form the lungs to peripheral tissues and are characterized by lack of internal organelles, including mitochondria, an adaptation that perhaps serves to increase their oxygen carrying capacity. Recently, Nix was identified as a protein that mediates elimination of mitochondria in reticulocytes (immature red blood cells; Schweers et al., 2007; Sandoval et al., 2008). Nix is a Bcl2-related protein with an atypical BH3 domain that is localized to outer mitochondrial membrane and is required for the elimination of reticulocyte mitochondria. *Nix*^–^^/^^–^ mice retain mitochondria in erythrocytes and develop anemia because of decreased survival of these cells ( Schweers et al., 2007; Sandoval et al., 2008). Studies of erythrocyte differentiation suggest that Nix is not required for induction of mitophagy *per se*, but for the engulfment of mitochondria by autophagosomes. Nix contains a cytoplasmic WXXL-like motif, which interacts with LC3 (the mammalian homolog of the yeast Atg8) and the GABA receptor-associated protein (GABARAP) *in vivo *and *in vitro *( Schwarten et al., 2009; Novak et al., 2010). Therefore, Nix appears to act as a receptor for targeting autophagosomes to mitochondria in a manner similar to the yeast Atg32 (**Figure 1B**). Nevertheless, despite the requirement of Nix in erythrocyte differentiation, treatment of reticulocytes with uncoupling agents induces mitophagy upon mitochondrial depolarization in a Nix-independent manner ( Sandoval et al., 2008). The mechanisms mediating Nix-independent mitophagy in reticulocytes remain unclear.

An additional important developmental role for mitophagy is the removal of paternal mitochondria in fertilized oocytes ( Al Rawi et al., 2011; Sato and Sato, 2011). Although, sperm contains mitochondria, which are transferred to the oocyte upon fertilization, only maternal mitochondrial DNA (mtDNA) is ultimately inherited. Two studies in *Caenorhabditis elegans *revealed that the autophagic pathway selectively degrades sperm mitochondria during the early stages of embryogenesis (**Figure 1C**). However, the signal that activates mitophagy, to selectively eliminate sperm-derived mitochondria remains unknown.

## MITOPHAGY IN NEURODEGENERATION

Neuronal cells typically require increased numbers of mitochondria, since most neuronal ATP is generated through oxidative phosphorylation. This high-energy demand is dictated by numerous neuronal processes, such as axonal transport of macromolecules and organelles, maintenance of membrane potential, loading and releasing neurotransmitters, and buffering cytosolic calcium. Therefore, neuronal survival and activity are critically dependent on mitochondrial integrity and functionality ( Rugarli and Langer, 2012). Mitochondria are highly dynamic organelles that constantly move and undergo frequent fission and fusion events. Several components of the fission/fusion machinery have been linked to various neurological diseases, underlying the significance of mitochondrial dynamics in neuronal homeostasis ( Alexander et al., 2000; Zuchner et al., 2004; Waterham et al., 2007). Recent studies have shown that fission/fusion dynamics not only sort out damaged mitochondrial components by distributing them throughout the mitochondrial network, but also fragment and isolate defective mitochondria prior to mitophagy ( Twig et al., 2008a,b). The interplay between mitochondrial dynamics and mitophagy is further underscored by the fact that excessive fusion prevents autophagic mitochondrial degradation ( Twig and Shirihai, 2011). Indeed, increased fusion protects mitochondria from massive degradation by starvation-induced autophagy ( Rambold et al., 2011). Therefore, modulation of mitochondrial dynamics, to increase fission or decrease fusion, facilitates isolation of damaged mitochondria and their subsequent elimination by mitophagy. Hence, mitochondrial damage and deregulation of mitophagy has been implicated in the onset and progression of several age-associated neurodegenerative diseases, such as Parkinson’s ( Schapira, 2011), Alzheimer’s, and Huntington disease ( Batlevi and La Spada, 2011).

### PARKINSON’S DISEASE

Parkinson’s disease is caused by loss of dopaminergic neurons in the substantia nigra, a region important for motor control and coordination. Loss-of-function mutations in PINK1 and/or PARK2 genes have been linked with the early onset of hereditary forms of Parkinson’s disease. The PINK1/Parkin pathway has been shown to regulate the elimination of damaged mitochondria through mitophagy ( Narendra et al., 2008, 2010). In addition, mtDNA mutations and/or deletions are more frequent in patients with Parkinson’s disease compared to age-matched individuals in the population ( Bender et al., 2006). Such mutations and/or deletions commonly appear and accumulate during aging in mitochondria of the substantia nigra neurons ( Kraytsberg et al., 2006). Consistently, loss of dopaminergic neurons in the substantia nigra that leads to the development of Parkinson’s disease correlates with mitochondrial damage accumulation in these neurons. Thus, excessive mitochondrial stress upon exposure to environmental toxins or defects in mtDNA, and the inability of the cell to eliminate damaged mitochondria through mitophagy, may contribute to Parkinson’s disease pathogenesis ( Ethell and Fei, 2009). However, mitophagy pathways have been characterized in non-neuronal cells, with neuronal mitophagy remaining a relatively obscure process. Some reports suggest that mitochondrial depolarization and respiratory deficiency do not induce Parkin recruitment in neurons ( Sterky et al., 2011; Van Laar et al., 2011). Other studies in neuronal cells indicate that Parkin is recruited to depolarized mitochondria and mediates mitochondrial elimination by mitophagy in a Parkin-dependent manner ( Wang et al., 2011; Cai et al., 2012). Thus, although mutations in PINK1 and Parkin have been associated with neurodegeneration in Parkinson’s disease, further work is needed to clarify if the PINK1/Parkin pathway regulates damage-induced mitophagy in neurons.

### ALZHEIMER’S DISEASE

Alzheimer’s disease is the most common age-associated neurodegenerative disorder, characterized by cognitive dysfunction and loss of memory, caused by neuronal cell death in cerebral cortex. Tissue sections from Alzheimer’s disease patient brains show distinctive intracellular neurofibrillary tangles and extracellular amyloid plaques composed of beta-amyloid derived from amyloid precursor protein (APP). While, the predominant hypothesis is that excess beta-amyloid leads to neuronal death, the mechanism that underlies pathogenesis is still unclear. Mitochondrial damage has been implicated in the development and progression of Alzheimer’s disease, since abnormalities in mitochondrial structure have been observed in afflicted individuals ( Baloyannis, 2006). Moreover, beta-amyloid fragments have been found to localize and accumulate within mitochondria ( Casley et al., 2002; Lustbader et al., 2004). In addition, the presence of autophagic vacuoles in neurons of Alzheimer’s disease patients further implicates cytoplasmic and organelle-specific degradation in disease progression ( Boland et al., 2008). In this context, mitophagy may have pivotal role in ameliorating, or defending against the development of Alzheimer’s disease through elimination of defective mitochondria, carrying cytotoxic beta-amyloid fragments.

### HUNTINGTON’S DISEASE

Huntington’s disease is an autosomal dominant neurodegenerative disease caused by the abnormal expansion of the cytosine, adenine, and guanine (CAG) repeats within huntingtin (Htt) gene. The severity of pathology correlates with the number of CAG repeats, the length of expansion ( Costa and Scorrano, 2012). Huntington’s disease is characterized by progressive motor dysfunction, as well as psychiatric and cognitive abnormalities caused by loss of cortical and striatal neurons ( Purdon et al., 1994). Expression of mutant Htt is associated with mitochondrial dysfunction both in patients and mouse models of Huntington’s disease. Decreased mitochondrial membrane potential, defects in mitochondrial calcium uptake, decreased respiratory function, reduced mitochondrial mobility and changes in mitochondrial structure are some of the observed mitochondrial defects in Huntington’s disease patients ( Bossy-Wetzel et al., 2008). Additionally, the peroxisome proliferator-activated receptor gamma coactivator-1a (PGC-1a), the master regulator of mitochondrial biogenesis, has been linked to metabolic and transcriptional defects in Huntington’s disease ( Weydt et al., 2006). Mitophagy may serve a protective function against neuronal loss in Huntington’s disease by eliminating damaged mitochondria. Consistent with this notion, recent findings indicate that Huntington’s disease pathology is associated with autophagic cargo recognition defects that lead to accumulation of damaged mitochondria in cytoplasm ( Martinez-Vicente et al., 2010).

## MITOPHAGY IN AGING

Mitochondrial dysfunction has already been correlated with aging. Mitochondria are the primary source of ROS, such as nitroxides, hydrogen peroxide, and superoxide anions ( Hekimi et al., 2011; Sena and Chandel, 2012). Aging particularly affects mitochondrial homeostasis, as ROS generation in mitochondria leads to mitochondrial protein and mtDNA damage. mtDNA is more sensitive and susceptible to oxidative damage due to lack of histones. mtDNA repair mechanisms are also less efficient or robust, compared to nuclear DNA. Accordingly, inhibition or abnormal synthesis of mitochondrial proteins exacerbates mitochondrial dysfunction. mtDNA mutations accumulate during aging, an event that has been correlated with age-related decreased autophagic activity ( Cuervo, 2008; Hubbard et al., 2012). In mammals, morphological and enzymatic mitochondrial defects occur during aging ( Navarro and Boveris, 2004). Therefore, it is possible that accumulation of damaged mitochondria could induce mitophagy to preserve cellular homeostasis. Studies in yeast have shown that deletion of the mitochondrial membrane protein Uth1 results in a selective defect in mitophagy and decreased lifespan upon nutrient deprivation ( Kissova et al., 2004). Caloric restriction is known to promote longevity from yeast to mammals. Given that caloric restriction induces autophagy, increased longevity may in part originate from enhanced elimination of dysfunctional mitochondria ( Yen and Klionsky, 2008). Further studies should clarify whether mitophagy is indeed involved in mediating part of the effects of caloric restriction on lifespan.

## CONCLUDING REMARKS

Although findings in diverse organisms indicate that the process of mitophagy requires the core autophagic machinery of cell, the initial signals that trigger and activate this selective type of autophagy, remain obscure. These signals appear to differ according to nutrient conditions, developmental processes, and damage-induced mitochondrial loss. Despite the fact that several proteins, such as ATG32, Nix, PINK1, Parkin have been identified as being critical for targeting mitochondria to autophagosomes, important information about the recruitment of these proteins to mitochondria and their interaction with the core autophagic machinery is lacking. Further investigation of the mechanisms mediating mitophagy will elucidate these key steps and shed light onto the link between mitophagy and aging. As a corollary, these studies are also likely to provide novel potential targets for therapeutic interventions against age-associated pathologies such as neurodegenerative disorders.

## Conflict of Interest Statement

The authors declare that the research was conducted in the absence of any commercial or financial relationships that could be construed as a potential conflict of interest.
